# Rediscovery of animality in the concept of sport: a posthuman perspective

**DOI:** 10.3389/fspor.2025.1688670

**Published:** 2026-01-09

**Authors:** Takuya Sakamoto, Yo Sato

**Affiliations:** 1 Institute of Health and Sport Sciences, University of Tsukuba, Tsukuba, Japan; 2 School of Education, Meisei University, Hino, Japan

**Keywords:** Derrida, digital technology, eSports, humanism, philosophy, sport, transhumanism

## Abstract

The concept of sport functions as an implicit premise in almost all sport-related research and practice, yet its definition itself is seldom subjected to critical scrutiny. Traditionally, sport has been characterized by elements such as playfulness, competitiveness, physicality, and organization. However, the concept is not universal; rather, it undergoes continual transformation in response to historical and social conditions, and many studies and practices have tended to overlook this aspect. This study, therefore, seeks to critically reassess the traditional concept of sport through the lens of posthumanism, a framework emblematic of contemporary digital technological society, and to propose a new perspective for redefining sport in the present era. Examining the contemporary phenomenon of esports from a posthuman standpoint reveals that various forms of physical violence and direct discrimination are preemptively mitigated by the digital mediation that characterizes these activities. In this sense, esports may be understood as a moral and distinctly more human form of sport. Such an understanding simultaneously exposes the extent to which conventional sport inherently contains inhuman dimensions, namely an element of “animality”, as an indispensable component. This insight aligns with arguments in sport ethics suggesting that existing sports have historically demanded forms of in/trans-human performance from athletes. According to Derrida, this dimension of animality has long been marginalized within anthropocentric modernity. From this vantage point, the present study's introduction of animality into the conceptualization of sport can be seen as a deconstructive gesture that challenges the modern, implicitly presupposed image of the human, typically Western, white, and male, and opens possibilities for reimagining sport as a genuinely inclusive and ecological cultural practice. At the same time, this perspective offers a novel vantage point for reconsidering various pressing issues in contemporary sport, such as genetic doping and binary gender eligibility regulations. In other words, situating animality at the core of sport's conceptual redefinition provides a critical foundation for rethinking the nature of sport in an increasingly digitalized society.

## 1 Introduction

### 1.1 Reconsidering the concept of sport

Today, the concept of sport serves as the foundation of all sporting activities worldwide. By relying on this concept, we are able to act as though various differences—such as region, language, and other minor distinctions—do not exist, and to discuss or conceive of sport as a common global subject. In other words, the concept of sport has long been regarded as self-evident. For example, in discussions of sport and development that have attracted attention over the past several decades (1), the meaning of sport may differ significantly between those on the development side and those on the receiving side. Nonetheless, these differences are often overlooked, and discussions and practices concerning sport development proceed on the assumption that there is a shared understanding of the concept. It should be noted that, as will be discussed in detail in Section 2, the term “sport (s)” as used in this paper refers to playful competitions involving physical activity with clearly defined rules, such as soccer and basketball.

The treatment of the concept of sport is prominent across academic research, particularly within the social (2) and natural (3) sciences. Internationally, most sport-related research belongs to one of these two fields. Beyond sport development, research in the social sciences—including sport business (4), sport management (5), sport education (6), and athletes' mental health (7), and the theory and practice of coaching (8)—is being actively conducted across countries, with new findings accumulating daily. Furthermore, scientific research in sport, ranging from medical and physiological studies on the relationship between sport or exercise and human health (9) to biomechanical research on athletic performance (10), has become indispensable in today's sport-centered society. Many of these studies and their outcomes have contributed, in no small part, to the realization of health and well-being for many individuals and for humanity as a whole beyond national borders (11). Furthermore, we should take note that, in any of these studies, the concept of sport is taken as a given.

In social and natural scientific studies of sport, the concept itself has rarely been questioned, primarily because such questioning would complicate the conduct of these studies. For researchers in these fields, the concept of sport functions as a premise, and examining its meaning or nature is deemed procedurally unnecessary. This questioning is similar, in most cases, even in interdisciplinary research and in many humanities studies related to sport. For instance, the study of sport history cannot begin without the presupposition of sport as its subject matter. Likewise, research and practices related to peace movements symbolized by Olympism are typically established only on the premise of the concept of sport (12). Furthermore, in order to elucidate the implicit knowledge or recognition underlying such diverse human practices, an empirical approach alone is not sufficient; rather, as will be discussed later, a philosophical approach can serve as an effective method.

The reason why such an approach is required is that the concept of sport is by no means unchanging. On this point, Kunihiko Karaki, a Japanese philosopher of sport, states, “Sport is not a fixed phenomenon but is changing. Sport has been historically created and developed, and it is possible that the content and form of sport, as we know them today, may disappear in the future (13, p. 13).” This fact is often overlooked in social and natural scientific studies. However, is it not precisely such implicit recognition and the issues it entails that are now emerging along with the globalization of modern sport? The challenges facing sport today are wide-ranging, encompassing disparities of various kinds (14), issues of gender (15), and instances of racial discrimination (16). Moreover, the rapid spread and integration of digital technology into modern sport compels us to reconsider the concept of sport that has long been taken for granted. As discussed later in this paper, the emergence of esports, in particular, has prompted renewed debates regarding the traditional concept of sport. Within the field of sport philosophy, these debates have become increasingly significant as esports have risen (17). In this paper, “esports” refers to conventional sports conducted through computer games or video games. As mentioned above, with reference to Karaki and others, this paper also refers to Japanese research in the philosophy and sociology of sport; however, since these are all based on discussions in Western philosophy, they are not necessarily related to Eastern thought. In addition, while this paper mainly refers to such discussions in Western philosophy, including those by Derrida, these debates possess a strong intent of self-critique. Therefore, it can be said that referencing them does not result in a biased argument.

### 1.2 Purpose and procedure

Based on the awareness of the issues described above, this paper examines the current state of the concept of sport from the standpoint of sport philosophy. More specifically, the purpose of this paper is to philosophically explore the retrospective question of what sport, which we take for granted, actually has been, and to clearly state the answer to this question. This endeavor is essential for actively transforming and even creating the meaning of the concept of sport, and at the same time, it will serve to prepare a theoretical foundation for considering how sport should exist in the future.

To achieve the above objectives, this paper will develop its argument along the following steps. First, Section 2 provides a brief overview of the evolution of the concept of sport. In particular, it refers to several definitions that have been the subjects of many studies, especially since the mid-20th century, when sport began to be treated in academic research. We will examine the definitions presented by Bernard Gillet, P.C. McIntosh, Paul Weiss, and Satoshi Higuchi, which have mainly been referenced in the field of philosophy of sport. The reason for deliberately including these decades-old pieces of literature as subjects of examination is that they have withstood philosophical criticism over a long period and continue to be cited in many studies to this day. In philosophical research, unlike in social or natural science research, it is necessary to deal with literature that has been established through historical evaluation rather than the most recent works. Although research in the philosophy of sport is by no means limited to the English-speaking world, due to the historical development of this field, literature from the English-speaking world has become the main focus. Through the examination of the above definitions, this paper demonstrates how the concept of sport has become taken for granted and no longer reflected over the past several decades, and, in doing so, points out the need for its revision. Next, in Section 3, as a clue to this update, we will focus on esports as one of today's characteristic phenomena surrounding sport. By examining esports from the perspective of posthumanism, we will highlight the uniquely “in/trans-human” characteristics found in conventional sports such as soccer or basketball that have existed in the real world. Furthermore, in Section 4, referring to the arguments of sport philosopher Shigeki Kawatani, we will clarify how this “in/trans-human” aspect inherent in conventional sport is closely connected to “animality.” Based on this view, in Section 5, this paper clarifies the fact that animality has been overlooked in traditional sport philosophy research and the reasons for this oversight, by referencing philosophical and anthropological discussions that these studies have relied upon, as well as Derrida's animal theory. Taking the above considerations into account, this paper will, in Section 6, finally discuss the significance and possibilities of newly including animality in the concept of sport.

This attempt of the paper is, in principle, positioned within the field of sport philosophy research. This is not a study that employs an approach centered on interpreting the thoughts of a single philosopher. Rather, this paper aims to update the very concept of sport by critically examining existing discussions surrounding the notion of sport and by exploring the issues that arise from this process — specifically, the expansion of our understanding regarding “in/trans-humanity.” If sport philosophy research like this differs from studies that address the ideas of a specific philosopher, the difference lies precisely in the fact that, as this paper attempts, the discussion is first grounded in individual phenomena within sport and unfolds based on that subject. In other words, this paper takes the very concept of sport as its subject, and in order to clarify its relationship with animality, it will pursue a philosophical approach, using various literature concerning sports and humanity, as well as the posthuman situation founded on digital technology, as reference points. Therefore, it is first necessary to examine how the concept of sport has been discussed up to now.

## 2 The evolution and stagnation of the concept of sport: a literature review

In this section, we would first like to briefly review matters that form the premise of this paper's discussion, namely, the evolution of the concept of sport. Of course, it is impossible to examine all references to the concept of sport. Therefore, we will focus on reviewing key discussions and debates in the philosophy of sport. By doing so, the overall picture of the concept of sport, which has often been presupposed as a premise, will become clearer.

Focusing on the evolution of the concept of sport, we find important discussions by scholars such as Gillet, McIntosh, and Weiss. While these works do not always address the conceptual characteristics of sport directly, they have frequently been referenced in subsequent studies. For example, one of the earlier and more widely cited definitions of sport by Gillet is as follows:

Three elements are required to recognize a certain physical activity as sport: play, contest, and intense physical exertion. As a result, we arrive at a more restricted but, at the same time, more noble notion of the word sport. The three conditions mentioned above are necessarily included in the definitions that conform to the concept of sport that we attempted to identify (18, pp. 12–13).

According to Hideki Nishimura (19, p. 765), this definition of sport by Gillet—namely, the definition that identifies play, contest, and intense physical activity as the necessary conditions for the establishment of sport—has been widely accepted globally. Looking back at the concept of sport and the substance of sport science research that followed, many indeed emphasize “intense physical activity” as an important requirement. This perspective, by assigning an important role to physicality in the concept of sport, enabled viewing sport as a cultural activity distinct from mere physical labor.

The three conditions for the establishment of sport proposed by Gillet are clear and easy to understand; therefore, they became widely accepted. However, Gillet's definitions do not necessarily capture the complex reality of sport. Modern sport has expanded in scope and form during the process of modernization. Regarding this point, McIntosh observed:

Sport, then touches human life at many pointsso many that it is difficult to define the concept or set limits to sporting activity. …In origin French, it designates any diversion from the sad or serious side of life. It covers activities ranging from mountaineering to making love, from motor-racing to playing practical jokes (20, pp. 10–11).

Thus, the range of sport as a concept is broad. As McIntosh notes, sport was originally rooted in human life, and in times when sport organizations were not as developed as today—that is, in eras when competitive sport was not common—the concept of sport was used in a broader sense. As is well known, this broad concept was refined over time as a more competitive sporting world emerged. Weiss, while reflecting on the nature of highly competitive sport and the ancient meanings derived from deportare, stated: “‘Sport’ both needs and deserves a steadier meaning than it has today (21, p. 133).”

Despite such concerns, attempts to redefine the concept of sport have been rare. Higuchi, a Japanese scholar of the philosophy of sport, provides one of the few exceptions. He states that “sport is physical activity (physicality) that, within a special context with different semantic connections from everyday life (playfulness), is based on artificial rules (organization), and includes competition with others and confrontation with nature (competitiveness) (22, p. 31).” Compared with Gillet's earlier definition, Higuchi's includes organization as a necessary condition. This suggests that the sport observed by Gillet in 1949 and by Higuchi 35 years later reflected significant changes in organizational structures, both internationally and domestically. If so, what kind of definition can we now give to the sport we observe today, about 40 years after Higuchi's proposal? Unfortunately, it appears that there have been no updates for as long as 40 years since Higuchi's definition. In fact, even in recent research by David Elstein (23), while the need to reconsider motor ability, namely physicality, one of the components of the concept of sport, as gross motor ability is pointed out, this does not fundamentally update the definition presented by Higuchi. Incidentally, Elstein also points out that the concept of sport is something that is constantly being updated.

As mentioned earlier, the nature of sport today is extremely diverse at multiple levels, making it increasingly difficult to define with precision. According to Frank McBride (24), the numerous existing definitions of sport highlight its diverse realities, and some have even argued that attempting to define it may be meaningless. However, isn't the lack of a proactive attitude to update the concept of sport in line with contemporary times making it difficult to fully understand and seek solutions to many of the issues currently faced in the sporting world, as mentioned earlier?

In response to this situation, as described above, this paper critically reconsiders the traditional concept of sport to capture its contemporary meaning and to update it. On this point, Karaki has remarked: “The task of clarifying the concept of sport primarily aims at critically examining the current situation (13, p. 12).” He further states, “If something that should never occur happens in the current state of sport, then the fundamental question of what sport trully inevitably arises (13, p. 12).” This underscores the necessity of reconsidering the concept of sport in today's context, where issues such as doping, violence, and various forms of discrimination have emerged. In other words, the concept of sport should be updated according to the times and social context, and this updating is made possible by critically reassessing the reality of sport, that is, through a philosophical approach.

## 3 Posthuman perspective

### 3.1 Posthuman and posthumanism

To critically reconsider the traditional concept of sport, this paper focuses on the notion of the posthuman as a defining feature of contemporary society. Today, this notion is often discussed in relation to modern technologies, including AI, exemplified by Ray Kurzweil's assertions that sparked debate on the “technological singularity (25).” However, the singularity itself has been widely criticized. For example, Hiroki Azuma, a Japanese thinker, critically examined the arguments of global historians such as Yuval Noah Harari and media artist Yoichi Ochiai, highlighting contemporary society's naive faith in technology and warning of its risks (26, pp. 138-167). The classical view of technology as merely a tool at human disposal has also been questioned. Dutch ethicist Peter-Paul Verbeek, for instance, points out that ultrasound technology used in obstetrics does not simply function to check the health of the fetus, but rather, there is a potential danger that its use morally compels us to use it (27). This suggests that technology shapes our ethics and morality, rather than remaining a neutral instrument. Thus, the relationship between humans and technology cannot be understood solely through the posthuman concept; it is far more complex.

Nevertheless, the posthuman perspective provides an important clue for rethinking that relationship. It fundamentally entails reexamining what it means to be human, thereby exposing the assumptions about humanity embedded in the traditional concept of sport. According to Rosi Braidotti, the posthuman is something that evokes both joy and anxiety regarding the disappearance of “Man,” which had been “the former measure of all things (28, p. 2).” In addition, this conception “raises serious questions as to the very structures of our shared identity –as humans– amidst the complexity of contemporary science, politics, and international relations (28, p. 2).” Posthumanism is the stance that seeks to understand the current situation in which the image of humanity, which has served as a premise for such modern and subsequent social systems, is being shaken.

It goes without saying that sport cannot exist without the people who practice it; therefore, it can be considered that a certain image of humanity is also associated with the concept of sport. If we examine the image of humanity that modern sport has implicitly assumed from the posthuman perspective, we can see that it has been that of a Western, white, male. The concept of the posthuman is precisely one that originates from considering the relationship between modern technology and humanity, based on a critique of the traditional view of the human that tacitly presupposed the conventional Western, white, and male as the norm (29, p. 5). In fact, prior to Braidotti, Nancy Katherine Hayles (30) had critically examined these premises and the associated traditional conception of humanity, pointing out both the possibilities and challenges of the posthuman concept. Even more interestingly, Donna Jeanne Haraway (31) sees such a symbol of the posthuman in the “cyborg,” portraying it as something that blurs the boundary between human and animal. This point is an issue that will later be connected to animality, which will be discussed in this paper. The observations by Braidotti mentioned above can be seen as a more contemporary update of such discussions. In this sense, it raises the question of how we should understand the human subject in modern contexts. Therefore, in this paper, we would like to position the concept of sport as an extension of such discussions surrounding contemporary society and examine its relationship with digital technology.

### 3.2 Esports as a more human form of sport

Building on this, the present study situates the concept of sport within contemporary debates about technology and society. The rapid development of digital technologies has transformed sport in practice. For example, Andy Miah's proposal of “Sport 2.0” illustrates this shift, exploring the convergence of sport and digital culture (32). Technologies related to referees' judgments and video technologies that support them are becoming an indispensable part of sport. At this intersection, sport and technology form one of the most debated topics today (33). In particular, ongoing discussions question whether esports can be considered sport (17). However, the focus of this paper is not on providing a binary answer to that question, but on examining the concept of sport itself, which underlies it. To advance this point, the study deliberately adopts the stance that esports constitutes a culture created by humans within its historical and social context, namely a kind of sport, and proceeds with the discussion from that standpoint. This perception and positioning of esports has some validity, as demonstrated by the IOC's Olympic Esports Series, held in Singapore from 22 to 25 June 2023 (34).

If we assume that esports are a form of sport, they could be described as an extremely “human” sport (35). In the virtual competitive space of esports, the physical violence traditionally problematized by sport ethics cannot occur. Furthermore, discrimination based on skin color is also avoided through avatars, and many individuals with disabilities who are unable to participate in conventional sports are given the opportunity to compete on equal footing with non-disabled players. In this sense, esports provide a safer competitive environment and may even be regarded as morally superior to conventional sports. As such, esports can be considered a more human form of sport, reflecting a direction of progress aligned with human aspirations.

Although issues caused by individual esports players are occasionally observed, these problems stem from participants themselves rather than the competitive form. Thus, criticisms regarding the human element in esports seem justified. If that is the case, from this single fact that esports is a more human sport, we can, in turn, question what conventional sports are. This question demands serious reflection. As will be discussed later, what emerges from this question is precisely the possibility that, while esports possess human qualities enabled by digital technology, conventional sport may lack these qualities, and, in fact, may even contain ‘inhuman’ elements that do not exist in esports. In other words, esports, emerging in a posthuman society, may be forcing us to confront the inhuman characteristics inherent in conventional sport. Precisely, this fact will require us to re-examine the traditional concept of sport and to create a new concept of sport. Braidotti's following observation about the posthuman society is suggestive for us who are tackling such challenges.

[T]he current scientific revolution, led by contemporary bio-genetic, environmental, neural, and other sciences, creates powerful alternatives to established practices and definitions of subjectivity. Instead of falling back on the sedimented habits of thought that the humanist past has institutionalized, the post-human predicament encourages us to undertake a leap forward into the complexities and paradoxes of our time. To meet this task, new conceptual creativity is needed (28, p. 54).

By further interrogating what is “human” and what is “inhuman” in a posthuman society, we can critically reconsider the existing concept of sport and, in turn, propose an answer to the question “What was sport?” In doing so, we can demonstrate the possibility of updating our traditional concept of sport and creating something new. Therefore, in the next section, we need to closely examine the discussions concerning inhumanity in conventional sport.

## 4 Humanity, inhumanity, and “animality as transhumanity”

What precisely do we call “sport”? As already noted, the clue to answering this question lies in the “inhumanity” brought to light by esports. Regarding the relationship between sport and inhumanity, Kawatani develops a distinctive and thought-provoking argument. Referring to his argument, this paper clarifies one characteristic of conventional sport.

Kawatani interprets the occurrence of various ethical issues in sport as an inevitability, grounded in the assumption that the essence of sport lies in competition. From this standpoint, practices such as doping and cheating inevitably arise from the drive to defeat others. Of course, Kawatani does not condone such actions. Rather, he questions why, given this essential characteristic, it is still necessary to “protect” sport from internal and external troubles. His response is telling: “If, to begin with, sport should be ‘protected’ from internal and external troubles, why is that? Isn't it because we can witness moments in sport where ‘humans transcend humans’ (Augenblick), in both a ‘good’ and ‘bad’ sense? (36, p. 66).” For Kawatani, the defining feature of sport is in the “moment, when humans surpass humans.” He elaborates further: “We watch sports not necessarily to see human-like humans, or ‘too human’ humans, but rather humans who are inhuman, humans who have transcended humanity (of course, there is probably an element of being drawn to something scary) (36, p. 66).” According to Kawatani, this “inhumanity” emphasized in this paper—the “human who is not human-like”—is discussed as equivalent to the “transhuman” that transcends humanity. But what exactly does “transhumanity” mean in the context of sport?

Kawatani further identifies the characteristics of conventional sport that encompass this transhumanity. In other words, he states that conventional “sport is not a culture that aims to domesticate the animality (naturalness) of humans to create truly human individuals—like ‘martial arts,’ which essentially involve holistic self-discipline—but rather a culture that prepares the foundation for humans to transform into a new kind of animal and to develop a new human nature (36, p. 66).” This observation aligns with the content discussed thus far in this paper, namely the differences identified between esports and conventional sport. Thus, while esports have manifested their humanity through technology, conventional sport may have shaped their characteristics precisely through “in/trans-humanity.” This view of humanity is related to the aforementioned posthumanism, but it can be said to be the position of “transhumanism”, which more directly emphasizes moving beyond the traditional mode of human existence. According to Kawatani, this transhumanity signifies a foundation for humans to transform into a new animal; in other words, it embodies animality embedded within conventional sport. Through Kawatani's discussion, the perspective this article has gained by critically examining the traditional concept of sport is precisely this animality.

Furthermore, regarding the role that this animality has played in conventional sport, Kawatani states: “The danger inherent in sport, which derives from various qualities such as ‘transcending human limitations’ (including everyday ethics), is literally two sides of the same coin with its appeal and cannot be separated. In other words, if danger is removed from sport, sport inevitably loses their appeal and identity simultaneously (36, p. 66).” Similarly, sport sociologist Masataka Kashihara notes the possibility that “animality” or “beastness” qualities have underpinned the appeal of traditional sport, and highlights the danger that these qualities are being compromised by modern digital technology (37, p. 313). From Derrida's standpoint, there is room for a more detailed examination of the similarities, differences, and relationships between animality (l'animalité) and bestiality (bestialité). This is because the concept of bestiality includes not only animal traits, but also the meaning of foolishness (38, p. 64). At the very least, however, these observations suggest that a major appeal of conventional sport can be seen as being guaranteed by its animality. And importantly, the point is that the animality inherent in such conventional sport has long existed latently without our awareness, and was brought to light with the advent of digital technologies in recent years, as symbolized by esports.

Thus, it is reasonable to perceive athletes not as beings that pursue humanity, but rather transhumanity, that is, animality, and to view sport as the world in which they live. This can be recognized, for example, in our abnormally heightened expectations for various “new records.” It is not uncommon for substantial rewards to be offered to break world or national records, regardless of the country or event. What this implicitly suggests is the single fact that we are never truly satisfied with simply watching excellent plays or performances, but are always, in sport, expecting something or someone that surpasses the current state. This is nothing other than the result of our desire to see athletes as beings that transcend the human, that is, as manifestations of animality, and the stage of sport on which they compete.

Based on the above discussions, it has been demonstrated that conventional sport inherently contains the characteristic of animality. However, even when reviewing previous discussions on the concept of sport, except for Kawatani, references to animality are scarcely observed, as far as our knowledge extends. Why has the animality aspect of sport not yet been discussed? More specifically, why has animality been overlooked in the study of sport philosophy? In the next section, we would like to explore this reason anew, using discussions on the traditional concept of sport as a clue. Through this examination, we hope to get closer to answering the question of what sport actually was.

## 5 The understanding of animality and its absence in the philosophy of sport

### 5.1 The privileging of the human body

To clarify why animality has been overlooked in the traditional concept of sport, it is useful to revisit past definitions of sport. As confirmed earlier in this article, the definitions of sport proposed by Gillet and Higuchi both focus on “physicality.” The emphasis on the human body is closely related to animality, as explained below, and it highlights the unique existence of the human body. In other words, perspectives on the human body differ from those on the animal body.

For example, sport philosophy researcher Tomihiko Sato identified the uniqueness of the human body while referring to the arguments of Austrian ethologist Konrad Lorenz (39, pp. 239-241). Sato emphasizes that the body is cultural, and not merely biological. Lorenz argued that human physical abilities have an overwhelmingly multifaceted nature compared to those of other animals:

Let us compare the purely physical abilities of humans with those of mammals of approximately the same size in terms of their various facets. Humans are neither feeble nor deficient creatures. For example, if given the three tasks of marching 35 kilometers in a day, climbing up a 5-meter hemp rope, and swimming 15 meters with 4 of those meters underwater to skillfully retrieve many items from the seabed, these are all things that even a bookworm who does not play sports at all could immediately manage. However, not a single other mammal could imitate a human and accomplish all of these tasks (40, p. 238).

Lorenz is not the only scholar to highlight the superiority of human physical abilities. For example, Arnold Gehlen, a central figure in philosophical anthropology, regarded humans as beings who lack many abilities compared with other animals. However, he argued that this very lack opens up broader possibilities. According to Gehlen, the world humans inhabit is “a world which is not an environment tailored to the human being, sparing in its stimuli, and accessible to him through his instincts, as is true of the environments of animals. The world is instead a field of surprises (Überraschungsfeld) in which man must first orient himself (41, p. 119).” That is why he says, “(T)he cultural world exists for man in exactly the same way in which the environment exists for an animal,” and therefore, he argues that “it is wrong to speak of an environment, in a strictly biological sense, for man (41, p. 29).” Furthermore, although “much too little attention has been given to the ability of the human being to enjoy a wide range of possibilities for movement, unknown among animal species,” “[t]he combinations of voluntary possible movements available to man are literally inexhaustible, the delicate coordinations of movements unlimited (41, p. 120).” In this manner, Gehlen also underscores the necessity and significance of focusing on the cultural and motor capacities of the human body—capacities absent in animals yet unique to humans.

These claims regarding the distinctiveness of the human body certainly provide one possible theoretical explanation for why humans created, widely practice, and have historically passed down the special culture of sport. Animals, after all, do not have such a culture, and therefore, sport is truly a uniquely human culture. The important point is that, when the strong connection between humans and sports culture is emphasized in this way, there is an implicit assumption that we humans are fundamentally different beings from other animals. In other words, this raises the question of whether, in emphasizing those kinds of arguments and the uniquely cultural aspects of human or sport, we may have, as an opposite reaction, overlooked the perspective of animality.

This issue is not limited to sport philosophy but also concerns Western philosophy more broadly, which has been the implicit framework of conventional research on the concept of sport. More precisely, the tendency to overlook animality in sport philosophy research reflects a wider challenge within Western thought. The clearest articulation of this is found in Heidegger's discussion of the relationship between animals and humans.

In *The Fundamental Concepts of Metaphysics*, Martin Heidegger explained the meaning of the world for humans: He said, “the stone is worldless, the animal is poor in world, man is world-forming (42, pp. 176–177).” As is clear from this observation, Heidegger considered animals to be deficient beings compared to humans, entities with limited relationships with the world. According to philosopher Koichiro Kokubun, Heidegger understood animals this way because he “held from the start the belief that humans are special and reasoned to fit this belief (43, pp. 281–283).” This observation may be useful in the context of sport, since the ideals of sport, symbolized by Olympism, place humanity at the center of their values. Thus, the greatness of sport is emphasized as a culture unique to humans, visible at every level of sporting activity. Familiar concepts such as fair play and sportsmanship exemplify this view. This is not to suggest that these principles are mistaken, but it is crucial to note that behind these ideals, something has been obscured—one of those hidden elements being animality.

### 5.2 Derrida's critique of the denial of animality

The fact that Heidegger, one of the most influential philosophers of the 20th century, held such an attitude toward animals indicates how animality has been perceived in Western philosophy. In fact, this disregard is not limited to Heidegger. As Derrida sharply observed, such a perception of animality has persisted throughout much of Western philosophy. Explaining why this view of animals has endured, Derrida, while referring not only to Heidegger but also to Descartes, Kant, Lacan, and Levinas, noted:

The experience of the seeing animal, of the animal that looks at them, has not been taken into account in the philosophical or theoretical architecture of their discourse. In sum they have denied it as much as [they have] misunderstood it. … It could not be the figure of just one disavowal among others. It institutes what is proper to man, the relation to itself of a humanity that is above all anxious about, and jealous of, what is proper to it (38, p. 14).

To summarize Derrida's argument: although we are humans, we do not account for the experience of being seen as animals, and thus we forget that we ourselves are animals. This disregard for, and forgetting of, animality would have been a natural consequence of the logos-centered tradition of Western philosophy. Animals, lacking language and therefore culture, were consistently understood as “world impoverished” beings, as per Heidegger.

Derrida further argues that this view persisted because animals were always regarded as “objects of sight.” As he notes, philosophers have acted “as if the men representing this configuration had seen without being seen, seen the animal without being seen by it, without being seen seen by it (38, p. 14).” Moreover, the perspective of animals as objects to be observed, and the issues it implies, is by no means limited to philosophical or abstract observations. As Patrick Llored notes, “Derrida's animal philosophy can primarily be understood as a bold operation of deconstructing the various boundaries between the real and symbolic (44, p. 29),” and therefore, the animal Derrida discusses is not limited to animals as physical beings, but also encompasses their symbolic significance.

Derrida's broad discussion of animality is also useful for understanding animality in the concept of sport. In practice, the most visible intersection of sport and animals is horse racing, dog racing, bullfighting, and cockfighting, all of which directly involve animals. On a symbolic level, the widespread use of animals as team names and mascots demonstrates even more vividly that animals have long served as “objects of observation” in the context of sport. This symbolic use, however, also conceals another reality: that we ourselves are animals. Interestingly, the adoption of animal names or mascots is less common among esports teams than among conventional sports teams.

Derrida's reflections on animality are closely linked to the growing need to rethink the equation “human = animal” in sport in today's digitally driven, posthuman society. His observations are particularly suggestive for understanding the relationship between sport culture, its concept, and animality.

[F]or about two centuries, intensely and by means of an alarming rate of acceleration, for we no longer even have a clock or chronological measure of it, we, we who call ourselves men or humans, we who recognize ourselves in that name, have been involved in an unprecedented transformation. This mutation affects the experience of what we continue to call imperturbably, as if there were nothing to it, the animal and/or animals (38, p. 24).

Although this remark is not explicitly about sport, Derrida's point regarding the changing perception of animality during this period is highly relevant. The very history in which we have developed, spread, and practiced modern sport culture as a distinctly human phenomenon coincides with this “two centuries” period. Thus, the history of the “civilization (45)” of modern sport, long taken for granted and codified in textbooks, is simultaneously a history of forgetting the animalistic dimension of sport.

This understanding of the animality inherent in such sport can also be endorsed by Elias's arguments regarding the “civilizing process (45).” What that discussion teaches us is that the process of humans taming their animality is what constitutes civilization, modernization, and the move toward nonviolence, that is, humanization. According to Elias, the background for the emergence of modern sport, mainly in Britain, lies in the establishment of a parliamentary system, which brought about the social realization of nonviolence. “This affinity is not accidental (45, p. 28).” The common thread is the realization of restraining one's fundamental impulses, and those who are unable to do so are pointed out as being unable to control their “animalistic need (45, p. 45).” In other words, in Elias's discussion of civilization, animality is seen as something that should be restrained, but this is by no means an argument to forget or erase the animality of humans. However, as this paper reveals, has this human civilization, through its alliance with today's digital technology, advanced not toward taming or domesticating this animality, but toward its disappearance and concealment? In the following section, we would like to present the findings revealed in this paper by exploring the answer to this question, and further discuss their possible applications.

## 6 Conclusion

### 6.1 The rediscovery of animality in the concept of sport

As a primary conclusion of this paper, it should first be emphasized that animality within the concept of sport has by no means disappeared, but rather has been forgotten throughout the history of modern sport culture. That is precisely why it has been rediscovered alongside the rise of digital technology, exemplified by esports today. Esports are truly a unique culture created only by humans, and this new form of human culture, in turn, highlights the characteristics of what we have traditionally called “sport.” In other words, the violence, brutality, and roughness that are lacking in esports were present in what we have traditionally called sport. At the same time, this suggests that we have implicitly expected these qualities in conventional sport. The rediscovery of animality within the concept of sport has only become possible through a critical reconsideration, from the perspective of posthumanism, of the human image that modern society has presupposed.

Based on the discussion up to this point in this paper, we would like to indicate its potential significance in today's sporting world. Regarding the relationship between digital technology and humans in contemporary sport, the already mentioned Kashihara uses the term “beastness,” akin to animality, and states the following.

[R]ather than sport taming technology, it should be seen that sport is tamed by technology that is empowered by capitalist desires. The subject that tames is not sport but technology. If this is the case, sport must instill fear in taming subjects. The beastness that sport should show technology is the primary impulse of play inherent in sport: the impulse to create rules and rewrite them through play (37, p. 313).

Today, the humanization of sport through its entanglement with digital technology does not tame animality but accelerates its concealment. We and contemporary sport are in that difficult situation. In such a situation, the rediscovery of animality signals the need to redefine the concept of sport and simultaneously urges a reconsideration of human nature within sport in an era where digital technology thrives. Issues such as gene doping, arising from advancements in genetic engineering technology, have extended far beyond the realm of sport and provoked complex problems of human bioethics (46). These problems are not confined to specialized areas such as sport ethics; rather, they must be addressed through interdisciplinary approaches that include psychology, sociology, medicine, and physiology. The animality discussed in this paper is expected to play an important role in such interdisciplinary research precisely because, as Llored earlier emphasized, it serves as a concept capable of bridging two dimensions of human existence: the symbolic cultural level and the biological reality.

In contemporary sport, the issue of eligibility to compete based on sex demonstrates this duality (47). Current gender controversies reveal the clash between the cultural and biological dimensions of athletes. Existing sports, which differentiate competitors by testosterone levels and rigidly uphold a binary gender system, may conflict most sharply with the cultural and gender norms of contemporary society. This paradoxically underscores the undeniable fact that what we have long called sport is situated between human cultural phenomena and biological phenomena. The current situation in sport is that our existing values are being shaken between these two aspects. In other words, the issues of gender and gene doping in contemporary sport are also confronting us with the presence of animality that has always existed within what we have traditionally called sport, yet has gone unnoticed. Therefore, in practice, when considering the introduction of technology in sport such as the further spread of video judging systems, we need to carefully examine not only the practical benefit of enhancing the accuracy of judgments, but also simultaneously assess whether such technology is accelerating our tendency to forget our animality.

As outlined above, this paper has demonstrated that although conventional sport undoubtedly possesses the characteristic of animality, it has not been properly recognized until now. The aim here is to propose adding animality as a component within the traditional concept of sport. This study has further illustrated that in discussions of sport, too much emphasis has often been placed on human exceptionalism. Consequently, esports have emerged, while unresolved biological and animal dimensions, such as those of genes and gender, have come to the forefront. Such problems will not be resolved in the future unless we learn to properly grasp, acknowledge, and effectively engage with our animality in sport. On the contrary, it appears that the deeper digital technology penetrates the field of sport, the more strongly our animality resists. Therefore, continuing to conceal the animalistic dimension as we have done until now is unlikely to be the correct approach. Moreover, as long as we remain human, we cannot eliminate it; rather, we must confront it directly. As Derrida observed:

It is too late to deny it, it will have been there before me who is (following) after it. After and near what they call the animal and with it—whether we want it or not, and whatever we do about this thing (38, p. 11).

If we consider this point in relation to the connections between conventional sport, technology, and animality as discussed in this paper, we can understand it as follows: in sport, we can never fully deny our own animality. This is because it is always, constantly behind us, near us, and with us. No matter how much we wish to overcome it, it never disappears. It is precisely for this reason that this animality occupies an essential position in the concept of sport. This is the conclusion reached by reexamining the concept of sport from a posthuman perspective.

### 6.2 Future issues and prospects

**Figure 1:**
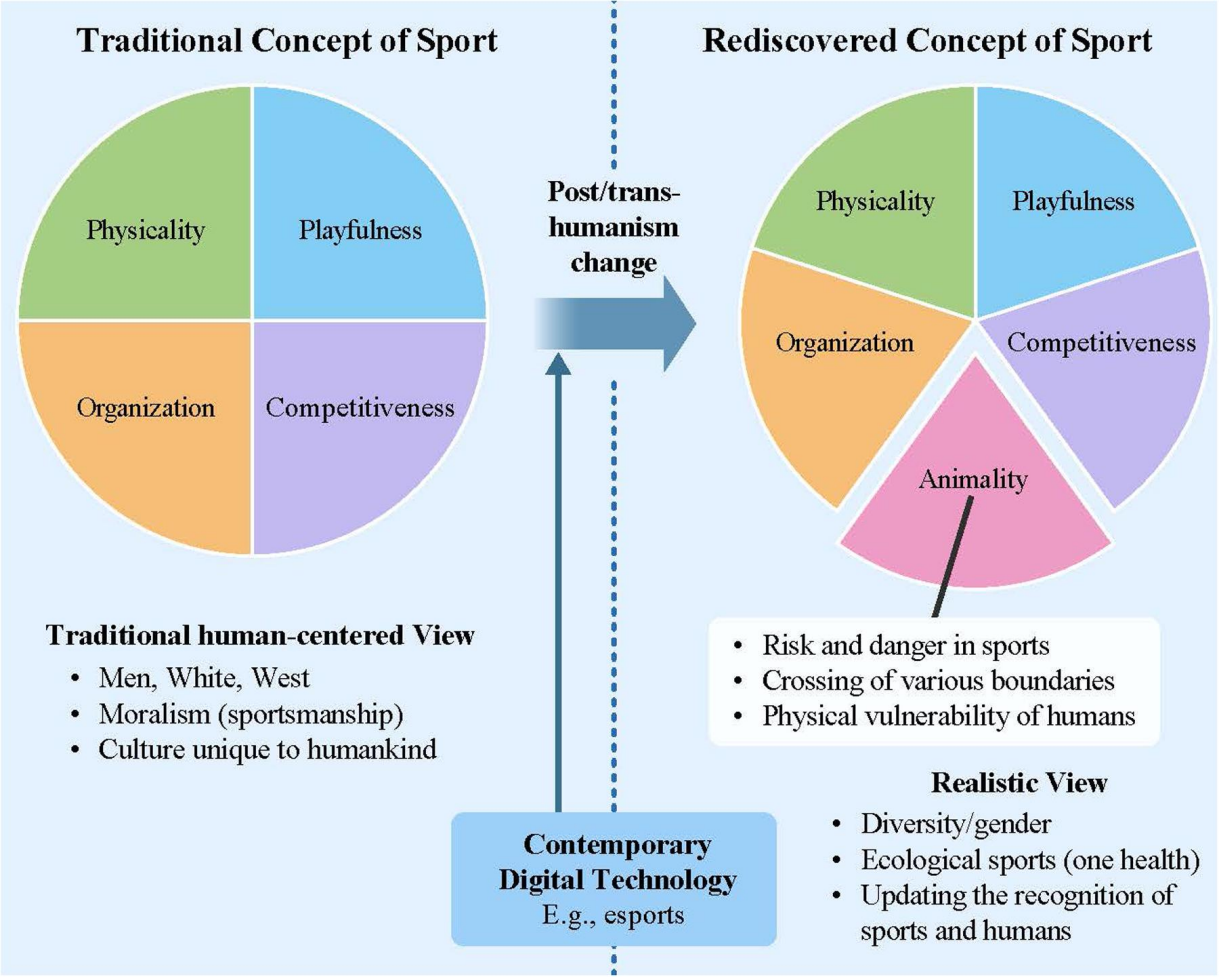
Rediscovery of animality in the concept of sport.

Finally, we would like to briefly outline the prospects of this paper. It is the applicability of the concept of animality. The concept of animality identified in this paper may be understood from several perspectives, enabling its application to more specific discussions in the future. For instance, rethinking the concept of animality through factors such as risk and danger in sport, the crossing of various boundaries, and the physical vulnerability of humans engaged in sport is expected to serve as an attempt to open up more concrete and practical possibilities for applying this concept. Such an attempt, by shifting the concept of animality from the symbolic level addressed in this paper to a more concrete level, could, for example, connect to contemporary issues such as “One Health (48),” which emphasizes understanding the health and well-being of humans, animals, and the environment as a unified whole, and may thus open up the possibilities for truly ecological sport (Figure 1).

In this paper, especially in the latter part, we developed our argument based on Derrida's discourse on animals. Regarding this point, it may be said that this paper has the limitation that most of the references cited are limited to Western philosophical sources, and it does not address approaches from knowledge systems in other regions. For example, as a recent study on animality and colonialism in Africa (49) suggests, the animality is not only an element of the concept of sport but may even have the potential to deconstruct the very nature of imperial/colonial modern sport. In addition, in the case of capoeira in Latin America, animality is found in its movements (50). This suggests that animality is not simply a concept originating in Western philosophy, nor is it limited to the context of modern sport. In other words, the focus on animality within the concept of sport is connected to the research theme of cross-cultural comparisons through the phenomenon of sport.

Furthermore, the interpretation of transhumanity in terms of animality is not exclusive to Derrida. For instance, the perspective of “animalization = dehumanization” claimed by Kawatani is, as Yuichiro Okamoto notes, also found in contemporary French philosophy beyond Derrida. According to Okamoto, “The ‘becoming animal’ that Deleuze and Guattari vividly described can be understood as the ‘dehumanization’ of humans.” Furthermore, “human desires are not completed solely within humans themselves but can connect with a variety of nonhuman entities, such as machines, animals, and computers. If that is the case, it can be said that the flow of desire ‘deterritorializing’ is directed toward ‘transcending humans’ (51, p. 138).” Thus, connecting this paper's argument to the discussion by Deleuze and Guattari may thus lead to a re-rediscovery of “animality” within the concept of sport. Such an attempt will not only deepen understanding of animality in sport but will also help reconsider extremely difficult ethical issues faced today—such as genetic doping and problems concerning eligibility based on the gender binary—from an entirely new perspective.

## References

[1] Hylton K. Sport Development: Policy, Process and Practice. 3rd ed. London: Routledge (2013).

[2] Smith B Caddick N. Qualitative methods in sport: a concise overview for guiding social scientific sport research. Asia Pac J Sport Soc Sci. (2012) 1:60–73. 10.1080/21640599.2012.701373

[3] Hopkins WG. Measures of reliability in sports medicine and science. Sports Med. (2000) 30:1–15. 10.2165/00007256-200030010-00001 10907753

[4] Kahn LM. The sports business as a labor market laboratory. J Econ Perspect. (2000) 14:75–94. 10.1257/jep.14.3.75

[5] Rudd A Johnson RB. A call for more mixed methods in sport management research. Sport Manag Rev. (2008) 13:14–24. 10.1016/j.smr.2009.06.004

[6] Hastie PA de Ojeda DM Luquin AC. A review of research on sport education: 2004 to the present. PESP. (2010) 16:103–132. 10.1080/17408989.2010.535202

[7] Ströhle A. Sports psychiatry: mental health and mental disorders in athletes and exercise treatment of mental disorders. Eur Arch Psychiatry Clin Neurosci. (2019) 269:485–498. 10.1007/s00406-018-0891-5 29564546

[8] Lyle J Cushion C. Sport Coaching Concepts: A Framework for Coaching Practice. 2nd ed. London: Routledge (2016).

[9] Kokko S. Sports clubs as settings for health promotion: fundamentals and an overview to research. Scand J Public Health. (2014) 42:60–65. 10.1177/1403494814545105 25416575

[10] Yeadon MR Challis JH. The future of performance-related sports biomechanics research. J Sports Sci. (2008) 12:3–32. 10.1080/02640419408732156 8158746

[11] Safai P. Sport, health, and well-being. In: Wenner LA, editor. The Oxford Handbook of Sport and Society. New York: Oxford University Press (2022). p. 379–398.

[12] Parry J. Sport and olympism: universals and multiculturalism. JPS. (2006) 33:188–204. 10.1080/00948705.2006.9714701

[13] Karaki K. Introduction. In: *The Physical Education Principles Subcommittee**,* editor. Concept of Sport (Principles of Physical Education II). Tokyo: Fumaido Publishing (1986). p. 9–20.

[14] Shimizu N. The Disparity in Children’s Sport. Tokyo: Taishukan Publishing (2021).

[15] Okada K Yamaguchi R Inaba K. Sport and LGBTQ+. Kyoto: Koyo Shobo (2022).

[16] Johnson W Jackson VP. Race and racism: the black male experience in sports. In: Hawkins BJ Carter-Francique AR Cooper JN, editors. Critical Race Theory: Black Athletic Sporting Experiences in the United States. London: Palgrave Macmillan (2016). p. 153–170.

[17] Parry J. E-sports are not sports. Sport Ethics Philos. (2019) 13:3–18. 10.1080/17511321.2018.1489419

[18] Gillet B. Histoire du Sport. Paris: Presses Universitaires de France (1949).

[19] Nishimura H. Play, sports, enjoyment. In: *Japan Society of Physical Education, Health and Sport Sciences**,* editor. New Encyclopedia of Sport Sciences. Tokyo: Heibonsha (2006). p. 764–766.

[20] McIntosh PC. Sport in Society. London: C. A. Watts & Co (1963).

[21] Weiss P. Sport: A Philosophic Inquiry. Carbondale, IL: Southern Illinois University Press (1969).

[22] Higuchi S. The Aesthetics of Sport. Tokyo: Fumaido Publishing (1987).

[23] Elstein D. On esports and competitive cooking: once more on the nature of sport. JPS. (2025) 52:98–113. 10.1080/00948705.2024.2408260

[24] McBride F. Toward a non-definition of sport. JPS. (1975) 2:4–11. 10.1080/00948705.1975.10654092

[25] Kurzweil R. The Singularity is Near: When Humans Transcend Biology. New York: Viking Adult (2005).

[26] Azuma H. Philosophy of Correctability. Tokyo: Genron (2023).

[27] Verbeek P-P. Moralizing Technology: Understanding and Designing the Morality of Things. Chicago: The University of Chicago Press (2011).

[28] Braidotti R. The Posthuman. Cambridge: Polity Press (2013).

[29] Katagiri M. Humans, AI, and Animals: Sociology of the Post-human. Tokyo: Maruzen Publishing (2022).

[30] Hayles NK. How We Became Posthuman: Virtual Bodies in Cybernetics, Literature, and Informatics. Chicago: University of Chicago Press (1999).

[31] Haraway DJ. Simians, Cyborg and Women: The Reinvention of Nature. New York: Routledge (1991).

[32] Miah A. Sport 2.0: Transforming Sports for a Digital World. Cambridge, MA: MIT Press (2017).

[33] Takaoka H. The differences between e-sports and regular sports: focusing on the characteristics of video games and body positioning. Taiikugaku Kenkyu. (2025) 70:541–556. 10.5432/jjpehss.33-25001

[34] International Olympic Committee. (2023) Available online at: https://www.olympics.com/en/esports/olympic-esports-series/ (Accessed November 12, 2025).

[35] Sakamoto T. Phenomenological critique of the esports experience: focusing on similarities with drone weapons. IJSHS. (2022) 20:110–116. 10.5432/ijshs.202141

[36] Kawatani S. Why do ethical issues arise in sport? Contemp Sports Rev. (2015) 32:56–66.

[37] Kashihara M. How Does the Technology Work in Sport? Kyoto: Sekai Shisosha (2021).

[38] Derrida J. The Animal That Therefore I Am. Mallet ML, editor. Wills D, translator. New York: Fordham University Press (2008).

[39] Sato T. Philosophizing Physical Education. Tokyo: Hokujyu Publishing (1993).

[40] Lorenz K. Das Wirkungsgefüge der Natur und das Schicksal des Menschen. München: R. Piper & Co. Verlag (1978).

[41] Gehlen A McMullan C Pillemer K. Man: His Nature and Place in the World. New York: Columbia University Press (1988).

[42] Heidegger M. The Fundamental Concepts of Metaphysics: World, Finitude, Solitude. Translated by William McNeill and Nicholas Walker. Bloomington, IN: Indiana University Press (1995).

[43] Kokubun K. Ethics of Leisure and Boredom. Tokyo: Ota Publishing (2015).

[44] Llored P. Jacques Derrida: Politique et Éthique de L’Animalité. Paris: Les Editions Vrin (2013).

[45] Elias N Dunning E. Quest for Excitement: Sport and Leisure in the Civilizing Process. Oxford: Blackwell (1986).

[46] Morioka M Ishii T Takemura M. Questioning Sports and Gene Doping: From Current Technology to Ethical Issues. Kyoto: Koyo Shobo (2022).

[47] Takemura M. Gender verification issues in women’s competitive sports: an ethical critique of the IAAF DSD regulation. Sport Ethics Philos. (2020) 14:449–460. 10.1080/17511321.2020.1775692

[48] Mijač S Slivšek G Džajić A. Deep ecology: contemporary bioethical trends. SEEMEDJ. (2022) 6(1):129–139. 10.26332/seemedj.v6i1.219

[49] Aderinto S. Animality and Colonial Subjecthood in Africa: The Human and Nonhuman Creatures of Nigeria. Athens, OH: Ohio University Press (2022).

[50] Delgado CF Munoz JE Durham NC. Everynight Life: Culture and Dance in Latin/O America. Durham, NC: Duke University Press (1997).

[51] Okamoto Y. Posthumanism: An Introduction to Philosophy in the Age of Technology. Tokyo: NHK Publishing (2021).

